# Proteomics analysis of colon cancer progression

**DOI:** 10.1186/s12014-019-9264-y

**Published:** 2019-12-28

**Authors:** Saira Saleem, Sahrish Tariq, Iffat Aleem, Sadr-ul Shaheed, Muhammad Tahseen, Aribah Atiq, Sadia Hassan, Muhammad Abu Bakar, Shahid Khattak, Aamir Ali Syed, Asad Hayat Ahmad, Mudassar Hussain, Muhammed Aasim Yusuf, Chris Sutton

**Affiliations:** 1 Basic Science Research, Shaukat Khanum Memorial Cancer Hospital and Research Centre, 7-A Block R-3, Johar Town, Lahore, 54000 Pakistan; 2 Institute of Cancer Therapeutics, University of Bradford, Tumbling Hill Street, Bradford, BD7 1BD UK; 3 Department of Pathology, Shaukat Khanum Memorial Cancer Hospital and Research Centre, 7-A Block R-3, Johar Town, Lahore, 54000 Pakistan; 4 Clinical Research Office, Shaukat Khanum Memorial Cancer Hospital and Research Centre, 7-A Block R-3, Johar Town, Lahore, 54000 Pakistan; 5 Cancer Registry and Clinical Data Management, Shaukat Khanum Memorial Cancer Hospital and Research Centre, 7-A Block R-3, Johar Town, Lahore, 54000 Pakistan; 6 Department of Surgical Oncology, Shaukat Khanum Memorial Cancer Hospital and Research Centre, 7-A Block R-3, Johar Town, Lahore, 54000 Pakistan; 7 Department of Internal Medicine, Shaukat Khanum Mmemorial Cancer Hospital and Research Centre, 7-A Block R-3, Johar Town, Lahore, 54000 Pakistan

**Keywords:** Colon cancer, iTRAQ proteomics, Orbitrap fusion, Biomarkers

## Abstract

**Background:**

The aim of this pilot study was to identify proteins associated with advancement of colon cancer (CC).

**Methods:**

A quantitative proteomics approach was used to determine the global changes in the proteome of primary colon cancer from patients with non-cancer normal colon (NC), non-adenomatous colon polyp (NAP), non-metastatic tumor (CC NM) and metastatic tumor (CC M) tissues, to identify up- and down-regulated proteins. Total protein was extracted from each biopsy, trypsin-digested, iTRAQ-labeled and the resulting peptides separated using strong cation exchange (SCX) and reverse-phase (RP) chromatography on-line to electrospray ionization mass spectrometry (ESI-MS).

**Results:**

Database searching of the MS/MS data resulted in the identification of 2777 proteins which were clustered into groups associated with disease progression. Proteins which were changed in all disease stages including benign, and hence indicative of the earliest molecular perturbations, were strongly associated with spliceosomal activity, cell cycle division, and stromal and cytoskeleton disruption reflecting increased proliferation and expansion into the surrounding healthy tissue. Those proteins changed in cancer stages but not in benign, were linked to inflammation/immune response, loss of cell adhesion, mitochondrial function and autophagy, demonstrating early evidence of cells within the nutrient-poor solid mass either undergoing cell death or adjusting for survival. Caveolin-1, which decreased and Matrix metalloproteinase-9, which increased through the three disease stages compared to normal tissue, was selected to validate the proteomics results, but significant patient-to-patient variation obfuscated interpretation so corroborated the contradictory observations made by others.

**Conclusion:**

Nevertheless, the study has provided significant insights into CC stage progression for further investigation.

## Introduction

Colorectal cancers (CRC) are the 3rd most common malignancies in the world. The global burden of CRC is expected to be more than 2.2 million new cases and 1.1 million deaths by 2030 [1]. In Asia, choice of unhealthy diet leading to obesity and metabolic syndrome, lack of public awareness on first choice of screening tests, poor public education for prevention, limited resources and support of healthcare authorities is resulting in increased susceptibility to CRC [2]. A positive relationship of family history, high fat diet, smoking and constipation exists among patients with a mean age of 41.47 ± 15.47 [3]. A cohort of 33 studies on over half a million subjects in the Asia-Pacific region concluded that other than body mass index and lack of physical activity (p < 0.05), height was strongly associated with CRC mortality such that an extra 5 cm in height was associated with 10% (95% confidence interval) additional risk, after adjustment for other factors. Diabetes and smoking increased the risk by 26% and 43%, respectively, while alcohol consumption, waist circumference and fasting blood glucose were not associated with CRC mortality [4].

Pakistan is situated in Southern Asia with distinct Punjabi, Sindhi, Pashtun, Baloch and Muhajir ethnic groups. The incidence of CRC is similar to developed Asian countries such as Japan, Republic of Korea and Singapore, but much lower than in Western developed countries [5]. Unfortunately, due to the absence of a national logging system for incidence, statistics on deaths due to cancers, survival rates cannot be estimated [6]. Information from individual health institutes, however, provides a valuable resource for Karachi, Sind [7], Rawalpindi and Punjab [8]. For example, the Cancer Registry at Shaukat Khanum Memorial Cancer Hospital and Research Centre (SKMCH&RC) operates to register cancer burden for the province of Punjab, and observed that CRC presents at an advanced stage and at a younger age (mean age 46.5 years) than other countries of similar wealth status [9]. The symptoms of CRC, such as rectal bleeding, persistent change in bowel habits, weight loss and continuous abdominal discomfort are often ignored due to embarrassment, but once addressed in a clinical environment are limited by the availability of doctors (7.8/10,000 population as per WHO observations) and resources (few screening centres in Punjab for a population of 30 million) to diagnose the disease through endoscopy and polyp histopathology. Consequently, a new front-line screening approach is required that can breakdown the cultural and economic barriers in Pakistan. The development of a biomarker-based diagnostic assay would enable greater acceptance of and accessibility to screening programs for CRC.

Colorectal cancer is a term used for the cancers of colon, rectosigmoid, rectum, anus and anal canal, which share similar histology however, distribution of cells and thickness of smooth muscles varies dependent on their function. Most of the pathology statistics are combined for CRC and do not provide regio-specific data. The colon is primarily responsible for the absorption of water and electrolytes while the rectosigmoid and rectum are for the storage of excreta for a short time. In SKMCH&RC, a total of 75,657 neoplasms (48.84% in males and 51.16% in females) primarily from Punjab were registered from 1994–2015, of which CRC ranked 5th most common (3530 cases) after breast cancer, leukemia, lip and oral cavity and non-Hodgkin’s lymphoma. Of the CRC cases 41.44% were colon cancers (964 in males and 499 in females), 7.19% were rectosigmoid (172 in males and 82 in females), rectal cancer 44.36% (1054 in males and 512 in females) and 6.99% of anus and anal canal (157 in males and 90 in females) (https://shaukatkhanum.org.pk/) [10].

Proteomics has been used previously to identify biomarkers of colorectal cancer though frequently using in vitro cell line models [11–13]. Those studies using biopsies often pooled samples from a number of patients from various sites (colon, rectum) and disease stages (metastatic and non-metastatic disease) to gain sufficient material for proteomics analysis. To support proteomics research of stage-specific (non-metastatic/node-negative and metastatic/node-positive) and site-specific (colon in our case) pooling of samples for specific protein profile, we pooled only colon tissues from exactly same stage (for non-metastatic colon cancer (CC NM). We pooled samples from patients who had primary disease in colon and were node-negative and for metastatic colon cancer (CC M), samples were pooled from patients who had tumors in colon and other organs (e.g. liver) of the body or were node-positive. Some studies have used matched, macroscopically normal margin around the borders of tumor for comparison of healthy and cancer tissues protein profiles, however, the margin may contain infiltrating tumor cells or cells in transition and therefore not be defined as normal tissue with certainty [14, 15]. For non-cancer (NC), we pooled colon lining tissues from non-cancer screening patients and for the benign, it was made sure that the patient has no malignancy.

One of the most complete studies to-date has been a label-free quantitative proteomics study of 64 colon cancer tissues and 31 rectum cancer tissues identifying 7526 proteins [16]. However, the molecular mechanisms underlying transformation of normal colon mucosa into malignancy and subsequent metastatic dissemination still remain unclear, and would benefit from a proteomics investigation of stage-specific disease development. To address this, and identify biomarkers indicative of disease progression, we performed a two-stage study. (a) iTRAQ-based proteomics analysis of disease progression using ethnically-, anatomically- and stage-specific, pooled colon biopsy protein extracts. (b) β-actin, Matrix metalloproteinase-9 (MMP-9) and caveolin-1 (CAV-1) western blot analysis of individual, stage-specific patient biopsies to compare with the proteomic analysis.

## Materials and methods

### Patients and samples

Ethical approval for this prospective study was obtained from the Institutional Review Board (registered with Office for Human Research Protections [OHRP], USA; IORG0004939) at SKMCH&RC, Lahore, Pakistan. A total of 98 tissue biopsies were obtained after getting consent from patients undergoing screening colonoscopy and/or invasive surgery from 2015–2019. Resected specimens were grossed by a pathologist and preserved either by fixing in formalin for embedding into paraffin wax for histopathological diagnosis or stored fresh at − 80 °C. The presence of cancer cells in the acquired specimen was confirmed independently by two clinical pathologists. For proteomic analysis, fresh frozen samples (n = 12) were selected from patients without prior chemotherapy and/or radiation and categorised as non-cancer, normal (NC), non-cancer non-adenomatous polyp (NAP), colon cancer, non-metastatic (CC NM) and colon cancer metastatic (CC M) (Additional file 1: Table S1a). For validation studies of selected targets, biopsies from an additional 86 patients without prior chemotherapy and/or radiation were collected (NC and NAP samples during colonoscopy and CC NM and CC M samples during surgery) for Western blot analysis (Additional file 1: Table S1b).

### Protein extraction

The samples were processed under identical conditions. Tissue biopsies (5 mm^3^) from minimum of two patients for each type of sample (NC, NAP, CC NM and CC M) were pooled. The protein extraction method was adapted from a previously described dual lysis buffer approach [17]. Each specimen was first homogenized in RIPA lysis solution (50 μL, PBS pH 7.4, 0.25% sodium deoxycholate, 0.1% SDS containing EDTA-free protease inhibitor cocktail) followed by vortexing for 30 min at room temperature (RT) and sonicated on ice for 20 s using a Status US70 sonicating probe (Philips Harris Scientific, Hyde, UK). The samples were then centrifuged at 13,4000 rpm for 20 min at 4 °C and liquid phase extracted to new tubes. Urea lysis buffer (50 μL, 7 M urea, 2 M thiourea, 4% w/v CHAPS, 50 mM DTT containing EDTA-free protease inhibitor cocktail) was added to the residual pellet, vortexed, sonicated and centrifuged as described above. The supernatant was combined with RIPA buffer extract. The urea lysis step was repeated on the residual pellet to extract all proteins giving a total volume of 200 μL. The protein concentration of samples was determined by Bradford assay (Sigma, Poole, UK). To a 70 μg aliquot of each protein extract, 1 mL of chilled acetone was added and allowed to precipitate overnight at − 20 °C. The precipitated contents were centrifuged at 13,400 rpm for 20 min at 4 °C, the supernatant discarded and the pellet used for proteomics preparation.

### Peptide preparation and iTRAQ labelling

Each protein precipitate (70 μg) was re-suspended in 8 M urea, reduced with 50 mM DTT for 20 min at 60 °C and then alkylated using 100 mM IAA for 20 min at RT, in the dark. Each mixture was diluted to reduce the urea concentration and digested with sequence grade trypsin (Roche Diagnostics, Burgess Hill, UK) (2.5 μL of 1 mg/mL prepared in 2 mM hydrochloric acid) was carried out overnight at 37 °C using a 1:10 trypsin-to-protein ratio (1:10 w/w). After digestion, peptide samples were lyophilized and re-suspended in 10 μL of 1 M tetraethylammoniumborohydride (TEAB) containing 0.1% SDS. Each digest was incubated with an iTRAQ 4-plex reagent (SCIEX UK Limited, Warrington, UK)—NC with iTRAQ 114, NAP with 115, CC NM with 116 and CC M with 117 (Table 1), for 90 min at RT. HPLC grade water was added to stop the reaction. The labelled peptides were then pooled, desalted on an Isolute C_18_ RP column (Kinesis Ltd., St. Neots, UK) and the 80% CH_3_CN, 0.05% TFA eluate lyophilized. The pooled iTRAQ-labelled samples (Table 1) were fractionated on a SCX column (Isolute SPE column, Kinesis Ltd.) using stepwise elution with 0.03, 0.06, 0.09, 0.12, 0.15, 0.18, 0.24, 0.3, 0.5, 0.7 and 1 M KCl buffer. The fractions, including flow-through containing non-bound peptides, were desalted through C_18_ RP cartridges, lyophilized and stored at − 20 °C.

**Table 1:** Pooled protein extracts for iTRAQ 4-Plex labelling

Pooled tissues from patients	Sample ID	iTRAQ label
n = 4	Non-cancer normal colon lining (NC)	114
n = 2	Non-adenomatous colon polyp (NAP)	115
n = 4	Colon cancer (non-metastatic) (CC NM)	117
n = 2	Colon cancer (metastatic) (CC M)	116

### HPLC-ESI MS/MS

The SCX fractions were analysed in duplicate on an UltiMate 3000 nano-HPLC—linked to an Orbitrap Fusion mass spectrometer (ThermoFisher, Hemel Hempstead, UK). Lyophilized fractions were resuspended in 0.1% formic acid (FA), 2 µL injected, and washed on a C_18_, 300 μm × 5 mm, 5 μm diameter, 100 Å Pep-Map precolumn (LC Packings, Sunnyvale, CA) before transfer to a C_18_, 75 μm × 50 cm, 2 μm diameter, 100 Å PepMap column (LC Packings) and peptides eluted with a linear gradient to 10−30% mobile phase B (80% acetonitrile, 0.1% FA) over 90 min, followed by 30–50% mobile phase B for 15 min. After washing in 90% solvent B for 10 min, the column was re-equilibrated for 20 min prior to the next injection. All data was acquired in data-dependent positive polarity mode with a spray voltage of 2200 V and ion transfer tube temperature of 275 °C. For MS, Orbitrap resolution was set at 120 K with scan range 350–1500 m/z, injection time of 50 ms. MS filter setting for parent ions; charge states from 2 to 7, dynamic exclusion duration was 70 and intensity threshold 5000. All MS/MS data was acquired in the Ion Trap with a quadrupole isolation window 1.2, CID collision energy 35%, with maximum injection time of 50 ms and AGC target 10,000. iTRAQ quantification was performed through MS^3^ in the Orbitrap; resolution of 30 K, mass range 110–500, 65% HCD collision energy and maximum ion injection time was 105 ms.

### Database searching

Proteome Discoverer 2.1 software was used to process Orbitrap Fusion data, with Mascot version 2.4 (Matrix Science, London, UK) search engine against the SwissProt database version 2016 (containing 552,259 human protein sequences). The search parameters used were: trypsin digestion, 2 missed cleavages, variable modification of methionine oxidation, fixed modifications of cysteine (carbamidomethylation) and iTRAQ (lysine and N-termini). A precursor mass tolerance of 10 ppm and fragmentation mass tolerance of 0.6 Da were selected. Non-redundant protein profiles were created by combining the search results from all 2D LC analyses using a confidence interval threshold set to a p-value < 0.05 (equivalent to a Mascot score of ≥ 22), and filtered to include only Master Protein Candidates with 2 or more PSMs and at least one peptide with Mascot score ≥ 22 (FDR for peptide and protein searches was < 1%). iTRAQ ratios were determined relative to the non-cancer control (NAP/NC − 115/114, CC NM/NC − 116/114, and CC M/NC − 117/114). The median ratio for the total complement of proteins for each comparison was determined, the ratios of the individual proteins normalised, converted to log_2_. Significantly up-regulated and down-regulated proteins for each experimental condition (NAP/NC, NM/NC and M/NC) were defined as those with ratio > ± standard deviation (SD) of log_2_ median. Protein-protein interactions analysis was performed using STRING (version 10.0, http://string-db.org/) for appreciably up- or down-regulated protein clusters.

### Western blotting

Protein extracts from individual tissue biopsies were prepared using RIPA lysis buffer as described above [17], 10 µg protein of each were analysed individually by SDS-PAGE (Bio-Rad Mini Gel, Hercules, CA) on 10% polyacrylamide gels and electroblotted to a polyvinyldifluoride (PVDF) membrane (Millipore; Immobilon-FL, 0.45-μm thickness) using a Mini Trans-Blot Electrophoretic Transfer Cell (Bio-Rad) [18]. Blotted membranes were blocked with 5% skimmed milk solution in 20 mM Tris; pH 7.5, 150 mM NaCl, 0.1% Tween-20 (TBST) for 45 min at room temperature, incubated with primary antibody [anti-goat human CAV-1 (R&D Systems, Abingdon, UK, 1:2000 dilution), anti-mouse human MMP-9 (R&D Systems, Minneapolis, USA, 4:1000 dilution or rabbit anti-human β-Actin (Abcam, Cambridge, UK, 1:1000 dilution)] overnight at 4 °C and washed 3 times with TBST. Protein extracted from one biopsy, for which there was an adequate supply, was selected for inclusion on each blot to enable inter-experimental normalization. Blots were incubated with horseradish peroxidase (HRP)-conjugated secondary antibody [polyclonal donkey anti-goat (R&D Systems; 1:1000), HRP conjugated polyclonal goat anti-mouse (R&D Systems; 1:1000) or HRP conjugated polyclonal goat anti-rabbit (Abcam; 1:10,000)], respectively, washed 3 times with TBST and once with TBS (without Tween 20). Proteins were detected using ECL solution (GE Healthcare, Little Chalfont, UK) and chemiluminescent exposures captured on a Syngene (Cambridge, UK) GBOX using default settings.

## Results

The main objective of this study was to look for proteins that play important biological roles in colon carcinogenesis and could be manipulated for their diagnostic value. Cancer patients were recruited based on TNM staging (Stage I, II, III, IV) in order to ascertain protein changes with disease progression (Table 1, Additional file 1: Table S1a). Stage I and II cancers are restricted to the primary site (colon) with no positive nodes and are referred to as non-metastatic (CC NM). In the case of stage III regional lymph nodes showed positivity for the presence of malignant cells confirming the disease spread while stage IV patients show malignant cells in secondary nearby organs as well. Both stage III and stage IV were referred to as metastatic (CC M), but all samples were taken from the primary tumors. We were also occasionally able to procure non-cancer normal colon tissues for comparison with matched NAP sample. The non-cancer status of the patients was confirmed by screening colonoscopy, taking a part of normal colon lining followed by its histopathology reports. Non-cancer patients were selected as a control group as there was less likelihood of mutated genes and the functional proteins would represent healthy profiles for comparisons with molecular signatures for cancer tissues. Tissue biopsies (5 mm^3^) from minimum of two patients for each type of sample (NC, NAP, CC NM and CC M) were pooled, from which an average of 70 µg of protein was extracted for proteomics analysis.

**Fig. 1:**
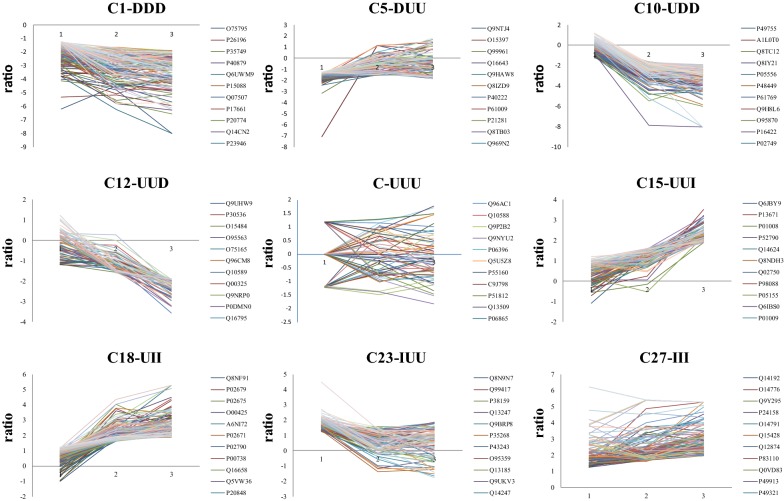
Selected clusters of functional significance 1: non-adenomatous colon polyp/non cancer 2: colon cancer (non-metastatic)/non cancer 3: colon cancer (metastatic)/non cancer Responses are defined as I: increased (ratio > ± 1 standard deviation (SD) of log_2_ median), D: decreased ratio (< ± 1 std dev of log_2_ median), U: unchanged (ratio = ± 1 standard deviation (SD) of log_2_ median). The colored lines represent proteins. Accession numbers for first 11 proteins in each cluster are shown in the legend

A total list of 2777 non-redundant proteins were detected with 2 PSMs, of which 2768 had 2 iTRAQ value (Additional file 2: Table S2), PRoteomics IDEntifications (PRIDE) PXD. Protein iTRAQ ratios were normalized to enable stage-related comparisons (NAP/NC, CC NM/NC and CC M/NC), and significantly up- and down-regulated proteins (+/− 1 log_2_ standard deviation respectively) were identified (Table 2). Proteins were clustered into 27 groups based in increased (I), unchanged (U) or decreased (D) for each of the three disease stages relative to normal colon, NAP/NC, CC NM/NC and CC M/NC. This analysis provided groups of protein associated with disease progression. As would be expected, the largest cluster comprising 59% of the total protein complement (Fig. 1, Cluster C14, UUU) exhibited no significant change in any of the three disease stages suggesting no roles in oncogenic processes or are modified post-translationally rather than via transcriptional activation /deactivation. Other clusters containing a large complement of proteins were those indicative of disease progression (Fig. 1). For example, 125 proteins were increased in all stages (Cluster 27, III, Fig. 1) relative to the NC control, representing early expression changes in colon cancer initiation and were sustained through to metastatic disease (Fig. 1). Whereas 121 proteins were increased in cancer tissues (CC NM and CC M, Cluster 18, UII, Fig. 1) which were associated with cancer progress and 78 increased only in the metastatic stage which were responsible for later oncogenic events (Cluster 15, UUI, Fig. 1). Encouragingly, the smallest clusters or those with no proteins, were those associated with sporadic responses, for example DDI (Cluster 3, Fig. 1) decreased in NAP and CC NM but increased in CC M). Within the main clusters, many of the proteins that changed in expression demonstrated functional importance, which were analysed by STRING analysis.

**Table 2:** Cluster analysis according to observed response modes of proteins in each of the diseased states relative to normal non-cancer tissue

Group	Response	No. of proteins	PPI enrichment, p-value
1	DDD	142	< 1.0e−16
2	DDU	36	4.88E−15
3	DDI	0	**–**
4	DUD	25	1.32E−09
5	DUU	93	*1.37E*−*06*
6	DUI	5	1
7	DID	0	**–**
8	DIU	0	**–**
9	DII	2	1
10	UDD	198	< 1.0e−16
11	UDU	56	2.81E−05
12	UUD	64	2.83E−12
13	UDU	0	**–**
14	UUU	1636	1.00E−16
15	UUI	78	3.86E−05
16	UID	0	**–**
17	UIU	48	4.25E−09
18	UII	121	< 1.0E−16
19	IDD	5	1
20	IDU	0	**–**
21	IDI	0	**–**
22	IUD	2	1
23	IUU	104	< 1.03−16
24	IUI	16	2.80E−05
25	IID	0	**–**
26	IIU	12	0.477
27	III	125	< 1.0E−16

Italic values indicate significance of p-values (p < 0.05). This means that proteins in the same group have enriched interactions indicating these are partially biologically connected

### Proteins changed in all stages of disease progression (clusters III and DDD)

There was a significant up-regulation of RNA metabolism and spliceosome-associated protein expression (p = 6.12e−15), cell cycle division proteins (cell division cycle 5-like protein, cell division cycle and apoptosis regulator protein 1, nuclear autoantigenic sperm protein, acidic leucine-rich nuclear phosphoprotein 32 family member A and B), and chromatin folding/remodeling proteins (high mobility group protein A1, B1, B2 and B3 isoforms and SWI/SNF-related matrix-associated actin-dependent regulator of chromatin subfamily E member 1) in all stages (III) relative to non-cancer indicative of enhanced replication, transcription and translation leading to cell proliferation in colon cancer stages (Additional file 3: Figure S1(i), Table 3a). Similarly STRING analysis of the proteins decreased in all disease stages (DDD) were very strongly associated with extracellular matrix organization (p = 8.86e−20) (Additional file 3: Figure S1(ii), Table 3b). Of particular note, 10 of the 17 collagen isoforms detected were decreased in all stages of colon malignancy and 6 others showing evidence of reduction compared to healthy controls. This represents a distinct difference in normal tissue comprising a high composition of extracellular matrix and stroma compared to tumors where the cellularity is more dense. Elastin and proteoglycans (basement membrane-specific heparan sulfate proteoglycan core protein, bone marrow proteoglycan, chondroitin sulfate proteoglycan 4 and proteoglycan 3), laminin alpha, beta and gamma subunits, decorin, biglycan, basigin and nidogen 1 and 2 also exhibited evidence of reduced levels compared to normal tissue.

**Table 3:** (a) Functionally significant proteins increased in all stages of disease or in cancer stages (III, UII) **(**b**)** Functionally significant proteins decreased in all stages of disease or in both cancer stages (DDD, UDD). Significantly changed expression (± 1SD) in italic cells

Accession	Description	Gene	MW (kDa)	Calc. pI	Mascot score	# Peptides Mascot	Coverage %	NAP/NC log2	CC NM/NC log2	CC M/NC log2
a										
Cell cycle progression, proliferation, DNA folding										
Q99459	Cell division cycle 5-like protein	CDC5L	92.194	8.18	41	2	3	*3.82*	*2.18*	*3.98*
Q8IX12	Cell division cycle and apoptosis regulator protein 1	CCAR1	132.739	5.76	106	3	3	*2.16*	*2.16*	*2.42*
P49321	Nuclear autoantigenic sperm protein	NASP	85.186	4.3	300	5	10	*1.32*	*1.84*	*2.43*
P39687	Acidic leucine-rich nuclear phosphoprotein 32 family member A	ANP32A	28.568	4.09	232	2	14	*1.67*	*2.06*	*2.34*
Q92688	Acidic leucine-rich nuclear phosphoprotein 32 family member B	ANP32B	28.770	4.06	367	2	14	*2.15*	*2.76*	*3.09*
P09429	High mobility group protein B1	HMGB1	24.878	5.74	1254	9	40	*1.74*	*2.27*	*2.80*
P26583	High mobility group protein B2	HMGB2	24.019	7.81	1066	7	25	*1.71*	*2.09*	*2.89*
O15347	High mobility group protein B3	HMGB3	22.965	8.37	450	4	28	*1.43*	*1.74*	*2.52*
P17096	High mobility group protein HMG-I/HMG-Y	HMGA1	11.669	10.32	213	2	33	*3.78*	*3.65*	*3.85*
Q969G3	SWI/SNF-related matrix-associated actin-dependent regulator of chromatin subfamily E member 1	SMARCE1	46.621	4.88	56	1	3	*1.80*	*2.37*	*3.16*
Matrix degradation										
P83110	Serine protease HTRA3	HTRA3	48.577	7.09	61	1	3	*1.31*	*1.74*	*2.83*
P80188	Neutrophil gelatinase-associated lipocalin	LCN2	22.574	8.91	417	3	23	*1.34*	*2.14*	*3.18*
P24158	Myeloblastin	PRTN3	27.789	8.35	658	4	28	*1.26*	*3.79*	*3.50*
P02788	Lactotransferrin	LTF	78.132	8.12	3542	30	49	*1.72*	*2.52*	*4.19*
P14780	Matrix metalloproteinase-9	MMP9	78.408	6.06	358	6	10	0.68	*3.38*	*3.69*
O75173	A disintegrin and metalloproteinase with thrombospondin motifs 4	ADAMTS4	90.139	7.85	26	1	1	0.60	*1.90*	*2.90*
P08246	Neutrophil elastase	ELANE	28.500	9.35	665	8	42	− 0.03	*1.87*	*2.82*
Cytoskeletal remodelling										
Q8NF91	Nesprin-1	SYNE1	1010.456	5.53	41	3	0	− 1.00	*1.80*	*3.14*
Q16658	Fascin	FSCN1	54.496	7.24	339	6	14	− 0.37	*1.84*	*3.24*
P59998	Actin-related protein 2/3 complex subunit 4	ARPC5	19.654	8.43	89	2	10	− 0.23	*1.61*	*2.54*
P55008	Allograft inflammatory factor 1	AIF1	16.693	6.24	566	1	14	− 0.19	*2.04*	*2.69*
P05109	Protein S100-A8	S100A8	10.828	7.03	2582	8	72	0.00	*3.76*	*2.38*
P06702	Protein S100-A9	S100A9	13.234	6.13	6645	7	63	0.19	*4.05*	*2.45*
P13796	Plastin-2	LCP1	70.244	5.43	1803	15	40	0.27	*2.68*	*2.07*
P40121	Macrophage-capping protein	CAPG	38.474	6.19	1382	9	35	0.33	*2.00*	*3.07*
Q96SB3	Neurabin-2	PPP1R9B	89.138	4.97	35	1	1	0.69	*1.73*	*3.42*
Q99439	Calponin-2	CNN2	33.675	7.33	668	5	24	0.75	*2.06*	*2.46*
Q12792	Twinfilin-1	TWF1	40.258	6.96	40	2	12	0.80	*2.04*	*2.35*
Q9UQB8	Brain-specific angiogenesis inhibitor 1-associated protein 2	BAIAP2	60.830	8.9	73	1	3	0.89	*2.41*	*2.46*
Q13177	Serine/threonine-protein kinase PAK 2	PAK2	58.006	5.96	173	3	16	1.09	*1.88*	*2.23*
Immunity/inflammation										
P02671	Fibrinogen alpha chai	FGA	94.914	6.01	2948	17	19	− 0.57	*2.36*	*3.84*
P02675	Fibrinogen beta chain	FGB	55.892	8.27	4950	28	61	− 0.95	*2.19*	*3.20*
P02679	Fibrinogen gamma chain	FGG	51.479	5.62	2304	18	49	− 0.96	*2.66*	*3.28*
P59665	Neutrophil defensin 1	DEFA1	10.194	6.99	358	2	19	0.34	*2.01*	*2.54*
P12838	Neutrophil defensin 4	DEFA4	10.497	8.02	54	1	18	1.19	*2.21*	*2.44*
b										
Extracellular matrix										
Q96P44	Collagen alpha-1(XXI) chain	COL21A1	99.307	8.32	76	1	1	*− 3.27*	*− 5.81*	*− 6.30*
P02462	Collagen alpha-1(IV) chain	COL4A1	160.514	8.28	1483	6	5	*− 2.70*	*− 2.71*	*− 2.94*
P39060	Collagen alpha-1(XVIII) chain	COL18A1	178.077	6.01	395	3	2	*− 2.00*	*− 3.09*	*− 2.75*
P08572	Collagen alpha-2(IV) chain	COL4A2	167.449	8.66	1983	13	12	*− 2.18*	*− 2.71*	*− 2.80*
P08123	Collagen alpha-2(I) chain	COL1A2	129.235	8.95	2714	9	8	*− 2.62*	*− 2.44*	*− 2.56*
P02452	Collagen alpha-1(I) chain	COL1A1	138.857	5.80	1814	9	9	*− 2.92*	*− 1.95*	*− 2.37*
Q05707	Collagen alpha-1(XIV) chain	COL14A1	193.394	5.30	894	20	16	*− 2.52*	*− 2.55*	*− 2.16*
Q14031	Collagen alpha-6(IV) chain	COL4A6	163.704	9.20	135	1	1	− 0.61	*− 2.46*	*− 3.94*
P12111	Collagen alpha-3(VI) chain	COL6A3	343.457	6.68	21068	100	41	*− 1.81*	*− 2.20*	*− 2.44*
P12110	Collagen alpha-2(VI) chain	COL6A2	108.512	6.21	5018	24	29	*− 2.15*	*− 1.85*	*− 1.99*
P12109	Collagen alpha-1(VI) chain	COL6A1	108.462	5.43	6769	25	37	*− 1.87*	*− 2.00*	*− 2.09*
P20908	Collagen alpha-1(V) chain	COL5A1	183.447	5.06	29	2	2	− 0.18	*− 2.20*	*− 2.24*
Q16363	Laminin subunit alpha-4	LAMA4	202.397	6.28	475	12	10	*− 2.72*	*− 2.05*	*− 1.98*
O15230	Laminin subunit alpha-5	LAMA5	399.479	7.02	1735	28	11	*− 2.89*	*− 3.34*	*− 2.30*
P55268	Laminin subunit beta-2	LAMB2	195.854	6.52	1451	27	23	*− 2.88*	*− 3.28*	*− 2.42*
P13727	Bone marrow proteoglycan	PRG2	25.189	6.76	637	5	30	*− 1.37*	*− 2.85*	*− 4.55*
Q6UVK1	Chondroitin sulfate proteoglycan 4	CSPG4	250.382	5.47	134	2	2	− 1.14	*− 2.98*	*− 2.95*
Q9Y2Y8	Proteoglycan 3	PRG3	25.389	4.81	222	3	16	− 0.33	*− 2.93*	*− 2.41*
P07585	Decorin 1	DCN	39.722	8.54	1574	13	51	*− 2.66*	*− 4.53*	*− 3.85*
P21810	Biglycan	BGN	41.628	7.52	1834	11	46	*− 1.73*	*− 1.91*	*− 1.89*
P14543	Nidogen-1	NID1	136.291	5.29	904	15	17	*− 2.65*	*− 2.98*	*− 2.43*
Q14112	Nidogen-2	NID2	151.158	5.29	640	11	13	*− 1.56*	*− 2.67*	*− 2.23*
P35613	Basigin	BSG	42.174	5.66	319	5	18	*− 1.71*	*− 2.02*	*− 2.22*
Cell adhesion and plasma membrane integrity										
P08648	Integrin alpha-5	ITGA5	114.465	5.77	678	10	13	*− 2.26*	*− 3.08*	*− 3.34*
P05556	Integrin beta-1	ITGB1	88.357	5.39	3088	16	25	− 1.21	*− 2.18*	*− 2.10*
P18084	Integrin beta-5	ITGB5	87.996	6.06	225	3	4	*− 1.28*	*− 2.18*	*− 2.03*
P23229	Integrin alpha-6	ITGA6	126.526	6.61	1739	23	25	0.18	*− 2.21*	*− 1.95*
P27216	Annexin A13	ANXA13	35.393	5.60	251	3	9	*− 1.57*	*− 2.85*	*− 3.33*
P20073	Annexin A7	ANXA7	52.706	5.68	225	3	8	*− 1.69*	*− 2.60*	*− 2.85*
P08133	Annexin A6	ANXA6	75.826	5.60	3275	30	50	*− 2.02*	*− 1.96*	*− 2.32*
P13688	Carcinoembryonic antigen-related cell adhesion molecule 1	CEACAM1	57.525	5.97	148	2	6	*− 2.64*	*− 4.05*	*− 3.02*
Q14002	Carcinoembryonic antigen-related cell adhesion molecule 7	CEACAM7	29.361	5.54	335	2	12	*− 2.05*	*− 3.26*	*− 3.91*
P09326	CD48 antigen	CD48	27.665	8.07	90	2	10	*− 1.39*	*− 4.10*	*− 4.48*
P13987	CD59 glycoprotein	CD59	14.168	6.48	428	4	25	− 0.88	*− 1.78*	*− 1.93*
P08962	CD63 antigen	CD63	25.619	7.81	74	1	9	− 0.48	*− 2.92*	*− 3.25*
P60033	CD81 antigen	CD81	25.792	5.29	184	3	25	*− 1.27*	*− 2.25*	*− 2.68*
P21926	CD9 antigen	CD9	25.399	7.15	475	3	21	0.09	*− 1.83*	*− 2.53*
Q99795	Cell surface A33 antigen	GPA33	35.609	4.93	694	9	37	− 0.72	*− 2.78*	*− 3.35*
P04233	HLA class II histocompatibility antigen gamma chain	CD74	33.494	8.44	296	3	9	− 0.17	*− 1.97*	*− 2.60*
P28907	ADP-ribosyl cyclase/cyclic ADP-ribose hydrolase 1	CD38	34.306	7.66	302	6	23	− 0.21	*− 2.11*	*− 2.29*
P04899	Guanine nucleotide-binding protein G(i) subunit alpha-2	GNA12	40.425	5.54	1216	10	36	*− 1.55*	*− 3.17*	*− 3.09*
Q9UBI6	Guanine nucleotide-binding protein G(I)/G(S)/G(O) subunit gamma-12	GNG12	8.001	8.97	31	1	26	− 0.11	*− 2.76*	*− 5.24*
P62873	Guanine nucleotide-binding protein G(I)/G(S)/G(T) subunit beta-1	GNB1	37.353	6.00	761	6	26	− 0.76	*− 3.82*	*− 4.44*
P62879	Guanine nucleotide-binding protein G(I)/G(S)/G(T) subunit beta-2	GNB2	37.307	6.00	527	6	26	− 1.01	*− 3.37*	*− 4.56*
P29992	Guanine nucleotide-binding protein subunit alpha-11	GNA11	42.097	5.69	364	3	15	*− 1.60*	*− 1.79*	*− 1.97*
Q14344	Guanine nucleotide-binding protein subunit alpha-13	GNA13	44.022	8.00	525	3	7	*− 1.43*	*− 2.28*	*− 3.42*
Q9HAV0	Guanine nucleotide-binding protein subunit beta-4	GNB4	37.543	6.00	116	3	13	*− 2.70*	*− 4.18*	*− 4.28*
Excretion, transport of metabolites and modification of xenobiotics										
O75795	UDP-glucuronosyltransferase 2B17	UGT2B17	61.055	8.54	895	8	14	*− 6.21*	*− 4.49*	*− 5.67*
P40879	Chloride anion exchanger	SLC26A3	84.451	8.69	91	2	3	*− 4.01*	*− 4.36*	*− 4.89*
Q6UWM9	UDP-glucuronosyltransferase	2A3	UGT2A3	60.215	7.96	234	4	*10*	*− 3.83*	*− 6.25*
Q14CN2	Calcium-activated chloride channel regulator	4	CLCA4	PE=1	1S0V1=.219	5.47	274	*5*	*6*	*− 3.59*
P54289	Voltage-dependent calcium channel subunit alpha-2/delta-1	CACNA2D1	124.49	5.27	59	2	3	*− 2.55*	*− 4.72*	*− 4.82*
P50443	Sulfate transporter	SLC26A2	81.609	8.38	102	2	4	*− 2.16*	*− 2.58*	*− 3.90*
Q96KA5	Cleft lip and palate transmembrane protein 1-like protein	CLPTM1L	62.189	8.56	68	3	8	*− 2.13*	*− 3.85*	*− 3.23*
O15438	Canalicular multispecific organic anion transporter 2	ABCC3	169.234	7.20	46	1	1	*− 1.95*	*− 2.72*	*− 2.90*
Q15041	ADP-ribosylation factor-like protein 6-interacting protein 1	ARL6IP1	23.347	9.32	27	1	5	*− 1.69*	*− 2.03*	*− 2.63*
O14936	Periphera plasma membrane protein CASK	CASK	105.056	6.43	28	1	1	*− 1.58*	*− 1.97*	*− 2.55*
P20020	Plasma membrane calcium-transporting ATPase 1	ATP2B1	138.668	6.04	123	5	4	*− 1.38*	*− 2.29*	*− 3.75*
P16662	UDP-glucuronosyltransferase 2B7	UGT2B7	60.655	8.31	618	3	7	*− 1.36*	*− 3.71*	*− 4.13*
P10301	Ras-related protein R-Ras	RRAS	23.466	6.93	217	3	15	*− 1.32*	*− 3.18*	*− 3.27*
Q8NE86	Calcium uniporter protein, mitochondrial	MCU	39.842	8.65	181	5	16	*− 1.32*	*− 2.48*	*− 3.15*
Q6UWW8	Carboxylesterase 3	CES3	62.242	5.62	137	3	10	− 0.46	*− 1.80*	*− 1.91*
P23141	Liver carboxylesterase 1	CES1	62.481	6.60	81	2	4	*− 2.12*	*− 1.78*	*− 1.90*
P50225	Sulfotransferase 1A1	SULT1A1	34.143	6.62	187	1	13	*− 3.21*	*− 3.38*	*− 4.38*
P0DMN0	Sulfotransferase 1A4	SULT1A4	34.174	6.01	240	2	17	− 0.97	*− 1.25*	*− 3.23*
P07099	Epoxide hydrolase 1	EPHX1	52.915	7.25	280	6	17	*− 2.41*	*− 2.29*	*− 2.42*
P37059	Estradiol 17-beta-dehydrogenase 2	HSD17B2	42.757	8.50	57	1	4	*− 2.52*	*− 3.78*	*− 3.76*
Q92506	Estradiol 17-beta-dehydrogenase 8	HSD17B8	26.957	6.54	402	4	20	0.57	*− 1.91*	*− 2.14*
P80365	Corticosteroid 11-beta-dehydrogenase isozyme 2	HSD11B2	44.098	9.28	453	6	22	*− 2.35*	*− 3.66*	*− 4.13*

The DDD cluster were also associated with cytoskeleton disruption (e.g. tropomyosin alpha-1, tropomyosin beta, tubulin beta-2A, myosin light polypeptide 6, myosin regulatory light polypeptide 9, myosin-11, filamin-C, desmin, smoothelin, leiomodin-1 and peripheral plasma membrane protein CASK) correlating with various structural changes in colon during disease progression, such as loss of cell contour associated with cell transformation and metastasis (Table 3b, Additional file 3: Figure S1(ii)). In addition, proteins responsible for colon mucosal function relating to excretion, transport of metabolites and modification of xenobiotics (UDP-glucuronosyltransferase isoforms 2A3, 2B7, 2B17, liver carboxylesterase 1, sulfate transporter, chloride anion exchanger, calcium-activated chloride channel regulator 4, cleft lip and palate transmembrane protein 1-like protein, canalicular multispecific organic anion transporter 2, ADP-ribosylation factor-like protein 6-interacting protein 1, mitochondrial amidoxime reducing component 2, mitochondrial calcium uniporter protein, plasma membrane calcium-transporting ATPase 1) were decreased, indication that the cells no longer function for their intended purpose in the colon.

An increase in serine proteases HTRA3, myeloblastin and lactotransferrin in all stages and metalloproteinases (MMP-9, ADAMTS4 and neutrophil elastase) in CC NM and CC M, supports degradation of the stroma surrounding the tissues as the tumor progresses. The action of these proteases may inhibit TGF-beta signaling indirectly through degradation of proteoglycans.

### Protein changes in malignant stages of the colon cancer (CC NM and CC M, clusters UII and UDD)

**Fig. 2:**
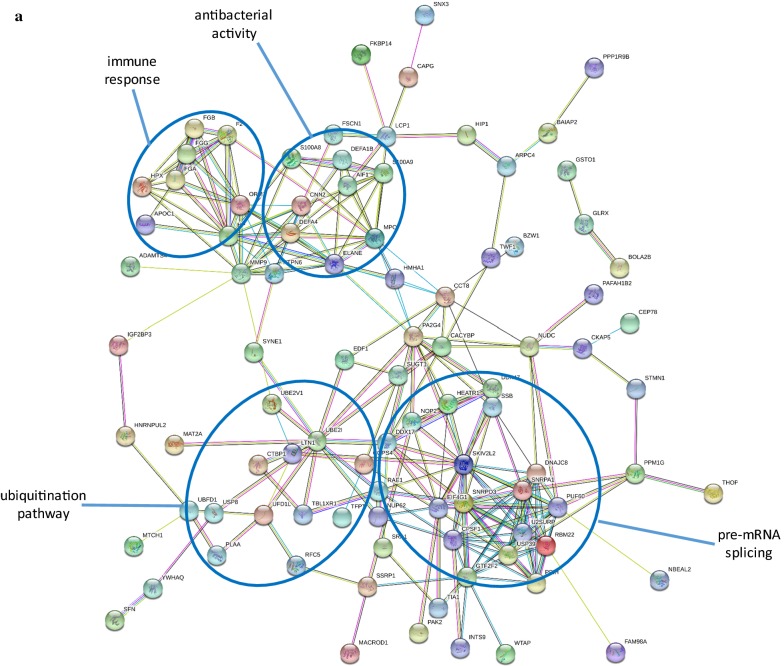

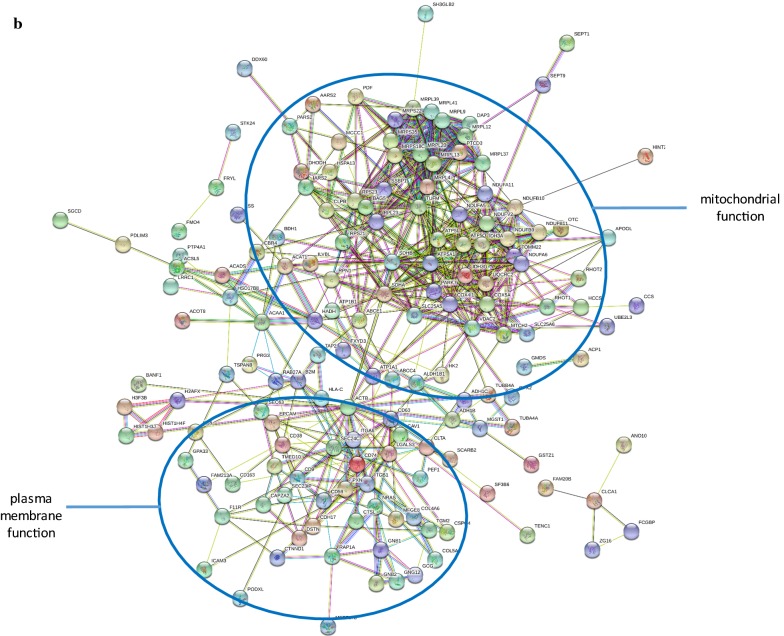
**a** STRING analysis of proteins increased in non-metastatic and metastatic stages of colon cancer (UII). **b** STRING analysis of proteins decreased in non-metastatic and metastatic stages of colon cancer (UDD). Proteins are shown as nodes and the color of each link defined the type of evidence available for the interaction between two proteins (e.g. blue: co-occurrence, black: co-expression, purple: experimental, aqua: databases, green: text mining and light blue is homology.)

In addition to matrix acting proteases, proteins increased in the cancer cluster (UII) were associated with inflammation/immune response and antimicrobial activity (neutrophil defensin 1 and 4, fibrinogen alpha, beta and gamma chains, prothrombin, protein S100-A8 and A9, cathepsin E, coiled-coil domain-containing protein 88B), cytoskeletal re-modelling (nesprin-1, fascin, actin-related protein 2/3 complex subunit 4, allograft inflammatory factor 1, plastin-2, macrophage-capping protein, neurabin-2, calponin-2, twinfilin-1, brain-specific angiogenesis inhibitor 1-associated protein 2, serine/threonine-protein kinase PAK 2) and 15 proteins involved in ubiquitin-linked protein degradation (Fig. 2a).

Those proteins that were decreased in cancer stages but not benign (UDD, PPI enrichment p < 1.0e−16), were noticeable associated with mitochondrial function affecting electron transport and the respiratory chain, citric acid cycle, ATP synthase, mitochondrial ribosomes and DNA replication, ADP/ATP transport and other metabolite trafficking (Fig. 2b). Overall this suggests a decrease in mitochondria composition within colon cancer cells (FDR = 1.67E−10). Glucagon was also one of the proteins increased in this group. Increased glucagon can result in hepatic gluconeogenesis contributing to metabolic syndrome cancer cachexia [19] leading to negative energy balance [20], weight loss and reduced food intake [21]. Elevated glucagon promotes release of glucose which stimulates tumor protein synthetic rate to double in colorectal cancer [22].

A group of proteins decreased in colon cancer non-metastatic and metastatic stages were associated with plasma membrane function indicating loss of cell–cell adhesion, cell junctions and cell-matrix interaction which was already in evidence in benign disease in the form of decreased G-protein-linked transmembrane signaling. Isoforms of G protein alpha, beta and gamma subunits, integrin alpha and beta subunits (α_1_, α_3_, α_5_, α_7_, α_x_, β_1_ and β_5_) and annexins (A1, A2, A4, A5, A6, A7, A11 and A13) exhibited a reduction in levels indicating a suppression of membrane signal transduction (see Table 3b). Integrins are stimulators of cancer cell proliferation and tumor angiogenesis [23] linked to the Ras-ERK pathway by CAV-1, tyrosine kinase Fyn and Shc [24] and integrin alpha-1 expression is directly regulated by oncogenic factor c-MYC [25]. N-Ras and R-Ras were both decreased in our proteomics dataset, along with proteins associated with transport vesicle and caveolae formation, including CAV-1.

### Protein changes in later stage of the colon cancer (CC M, clusters UUI and UUD)

Those protein uniquely increased in tumors that have advanced to a metastatic stage (UUI, 78 proteins), indicating an escalation of immune/inflammation response (complement component C6, factor H, syntenin-1, apoptosis-associated speck-like protein containing a CARD, protein canopy homolog 3) and ubiquitin-associated protein turnover (ubiquitin-conjugating enzyme E2 N, ubiquilin-1, -2 and -4, TP53-binding protein 1 and centrosomal protein of 131 kDa). Ubiquilin proteins play a role in LC3-mediated production of autophagosomes, a process indicative of the harsh microenvironment of solid tumors starved of oxygen and nutrients, from which select cancer cell may well survive to spread by metastasis (Additional file 3: Figure S1 (iii)) [26].

Those proteins decreased only in late stage colon cancer indicated further losses in mitochondrial function and membrane transport activity (Additional file 3: Figure S1(iv)). In addition, mucin-2, which is a highly abundant component of colon mucus layers was decreased and suggestive of a massive disruption of normal tissue morphology as these advanced tumors invade the surrounding organs. Interestingly, there was an inverse response in the expression of mucin-5AC, which is normally detected in gastric and respiratory tract mucosa.

### Protein signatures specifically related to benign disease (NAP, clusters IUU and DUU)

Increased protein signatures, uniquely associated with benign disease (non-adenomatous polyps) were strongly coupled with transcriptional (spliceosomal) and translational processing (ribosomal) (FDR = 8.13E−25) (Additional file 3: Figure S1(v)). No clear functional profiles was derived from the analysis of the decreased proteins, though many components were linked to neutrophil degranulation (FDR = 4.01E−07) (Additional file 3: Figure S1(vi)).

### Caveolin-1 and MMP-9 expression

We aimed to validate a protein identified with as low as ≤10 peptides in our ESI-MS data and had already been associated with colon cancer progression. So we selected caveolin-1 (CAV-1) from the cluster C10 (Fig. 1) UDD (unchanged between benign and non-cancer tissues but decreased in both non-metastatic and metastatic and tumor as compared to non-cancer tissues) and Matrix metalloproteinase-9 (MMP-9) from the cluster C18 (Fig. 1) UII (unchanged between benign and non-cancer tissues but increased in both non-metastatic and metastatic and tumor as compared to non-cancer tissues) for further investigation. In so doing we wished to determine if CAV-1 and MMP-9 have the potential to be a suitable biomarker. There is contradictory evidence of the expression changes of CAV-1 in different cancer tissues [27–31], yet clear pivotal role in different oncogenic processes. Although not used diagnostically, the role of MMP-9 has been well described in CRC invasion and metastasis, enabling tumor growth and cancer cells to escape into circulation [32–35].

**Fig. 3:**
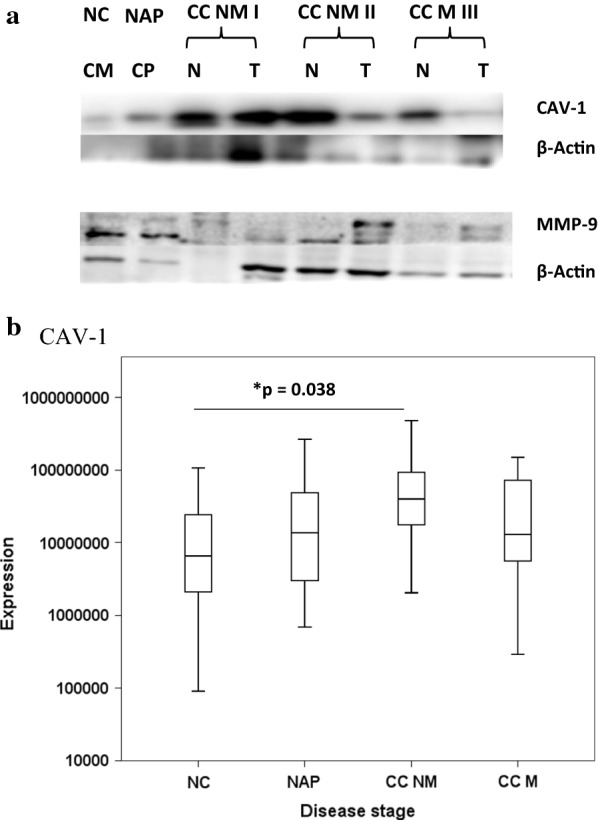
**a** Western blot expression of CAV-1 (21–24 kDa) and MMP-9 (65–92 kDa) identified in diseased and non-cancer colon tissues NC: non-cancer normal colon lining, *NAP* non-adenomatous colon polyp, *CC NM* colon cancer (non-metastatic), *CC M* colon cancer (metastatic), *I–III* TNM staging according to AJCC 8th Edition guidelines. **b** Box and whisker plot of the CAV-1 expression by western blotting versus normal colon or disease stage

We screened the expression of these proteins on discrete and unique patient samples ranging from tissues from non-cancer (screening patients) to benign/non-malignant polyps and poorly- to well-differentiated colon cancer tissues. We intended to analyse our patient population (Asian/Pakistani) and establish CAV-1 and MMP-9 significance in colon cancer. CAV-1, MMP-9 and β-actin (loading control) were analysed by western blotting of protein extracts from an independent cohort of 86 patients with well characterized normal colon mucosa, benign polyps and tumors from stage I-IV tissues histopathologies (Fig. 3a, Additional file 4: Figure S2). Our results indicate CAV-1 is expressed in all tissue types in various degrees. Normal tissues from non-cancer patients (Fig. 3a: NC, NAP) expressed CAV-1 and MMP-9. The slight increase in expression of CAV-1 in CC NM I T compared to CC NM I N (Fig. 3a) shows a different response to that determined by proteomics analysis where a decrease in tumor was observed as compared to the non-cancer tissue (UDD). However normal tissues from cancer patients (Fig. 3a: CC NM II N and CC M III N) expressed higher CAV-1 than the matched tumor tissues (Fig. 3a: CC NM II T and CC M III T). This decrease in CAV-1 expression in tumor tissue suggests that loss of CAV-1 is required for the disease to establish which relates to clinical outcome. Overall the findings of the study reflect patient-to-patient variations. This warrants further investigation to determine if CAV-1 is a biomarker of early colon cancer or an indicator of abnormal growth in colon mucosa. Analysis of the cohort (Fig. 3b, Additional file 5: Table S3a), indicates that the trend of expression of CAV-1 is increasing from NC to CC NM (p<0.038, Additional file 5: Table S3c), but then decreases in CC M. Similar properties for β-actin were observed. The MMP-9 (Fig. 3a) shows a similar response to that determined by proteomics analysis where an increase in tumor was observed as compared to the non-cancer tissue (UII). Tumor tissues from cancer patients (Fig. 3a: CC NM II T and CC M III T) expressed higher MMP-9 than the matched normal tissues (Fig. 3a: CC NM II N and CC M III N). This increase in MMP-9 expression in tumor tissue suggests that increased expression of MMP-9 is required for the disease to establish or spread. Overall the findings of the study reflect patient-to-patient variations. This warrants further investigation to determine if CAV-1 and MMP-9 are biomarkers of early colon cancer or an indicator of abnormal growth in colon mucosa.

## Discussion

Many of those proteins that were significantly changed in this investigation, have formerly been associated with colon cancer, including HLA class II histocompatibility antigen gamma chain (CD74) [36], carcinoembryonic antigen-related cell adhesion molecule-7 (CEACAM-7) [37], glutathione S-transferase omega-1 (GSTO1) [38], matrix metalloproteinase-9 (MMP-9) [35], hepatoma-derived growth factor (HDGF) [39], lactotransferrin (LTF) [40], cytoskeleton-associated protein 5 (also known as colonic and hepatic tumor overexpressed gene protein) [41] and DNA (cytosine-5)-methyltransferase 1 [42]. CD74 was previously shown to be decreased in cancer samples based on mRNA levels (NAP/NC ratio log_2_ − 0.169, CC NM/NC − 1.966 and CC M/NC − 2.601) and CEACAM-7 (NAP/NC ratio log_2_ − 2.054, CC NM/NC − 3.259 and CC M/NC − 3.911) based on immunohistochemistry staining. GSTO1 expression was unchanged in non-adenomatous, but significantly elevated in non-metastatic and metastatic states (NAP/NC ratio log_2_ − 0.022, CC NM/NC 1.854 and CC M/NC 2.452). GSTO1 knock-down by the inhibitor C1-27 in colon cancer cell line HCT116 resulted in down-regulation of Dickkopf-related protein 1 (DKK1), thombospondin 1 (THBSS1) and CAV-1 [43]. MMP-9 (NAP/NC ratio log_2_ 0.677, CC NM/NC 3.379 and CC M/NC 3.691), which is responsible for extracellular matrix degradation exhibited an even more profound response. Two anti-apoptotic proteins HDGF (NAP/NC ratio log_2_ 1.349, CC NM/NC 2.237 and CC M/NC 3.244) and LTF (NAP/NC ratio log_2_ 1.724, CC NM/NC 2.521 and CC M/NC 4.186) were increased in all disease conditions. HDGF is associated with CRC progression with cellular proliferation, migration, invasion, and tumorigenesis noticeably decreased in HCT116 and HT29 in vitro and in vivo HDGF knockdown models [39, 44]. LTF knockout resulted in inflammation-induced colorectal dysplasia in mice probably by inhibition of NF-κB and AKT/mTOR signaling suggesting a protective role in colorectal inflammation**-**related malignant transformation [40].

Metabolic functions of mitochondria are instrumental in tumor anabolism, oxidative stress, Ca^2+^ homeostasis and cell death. [45]. Accumulation of dysfunctional mitochondria generates increased tumor-promoting reactive oxygen species (ROS) and tumorigenic signals [46]. Clearance of dysfunctional mitochondria via mitophagy is critical for cellular fitness [47]. A major trigger for mitochondrial clearance in tumor cells is mitochondrial membrane depolarization and hypoxia, activating PTEN-induced putative kinase 1 (PINK1)/Parkin pathway or through Bcl-2 [46]. Elevated mitochondrial fission by dynamin-related protein-1 (Drp1) recruitment leading to impaired cancer cell growth suggests its importance in tumorigenesis [48].

CAV-1 moderates lipid trafficking and is known to be up-regulated in drug resistant cancer cell lines [28] and in colon cancer, histone modifications bring about the genetic drift in CAV-1 gene regulation instead of DNA methylation [49], which agrees with the observations by Western blot analysis. CAV-1 plays an important role in cell migration and is a regulator of the K-RAS oncogene in colon carcinogenesis. It was demonstrated that colon tumor cells harboring K-RAS mutations had higher levels of CAV-1-1 mRNA levels involving the AKT pathway [50]. Paradoxically, decreased expression of CAV-1 has also been indicated as a potential prognostic factor for CRC [31, 51], which correlated with the proteomics results observed in this study with significant decrease from CC NM to CCM, but not NAP (NAP/NC ratio log_2_ − 0.712, CC NM/NC − 2.193 and CC M/NC − 2.466). CAVs are structural proteins forming 50- to 100-nm invaginations of the plasma membrane called caveolae that function as regulators of signal transduction. CAV-1 levels are positively correlated with tumor stage/grade in a number of cancer types and regulates multiple cancer-associated processes including cellular transformation, tumor growth, cell migration, cell death and survival, multidrug resistance and angiogenesis [52]. CAV-1 gene endogenous expression or re-expression may also reduce pancreatic carcinoma cell invasion probably through Erk-MMP signal pathway [53]. Furthermore, expression of CAV-1 in the ovarian carcinoma cell line OVCAR-3, resulted in suppression of tumor cell survival in vitro, suggesting that the CAV-1 gene is likely to act as a tumor-suppressor gene in human ovarian epithelium [54]. CRC patients with high stromal CAV-1 had a good (92%) 5-year survival rate, in contrast to patients with moderate levels or absent CAV-1 [54]. Overall, the complex role of CAV-1 in cancer explains the divergent results we have observed reflecting patient-to-patient variation in oncogenic events. Interestingly, β-actin was also observed to be decreased in colon proteomics results, whilst Western blotting results indicate a rise with early stage disease. As with CAV-1, there was significant patient variation, which may reflect tissue heterogeneity independent of cancer stage. One of the challenges of the studies using β-actin as a loading control is that β-actin is a protein itself and its expression varies from patient-to-patient just as with several other proteins. We reported our observations which emphasize the fact that alternate loading control strategies should be followed instead of selecting patient proteins which are all altered in case of the colon cancer disease. We have screened a large number of patients tissues (n = 86) using western blotting that shows expression variations in different patients. This study is the first to report CAV-1 expression in non-malignant and malignant colon-specific tissues both by proteomics and by classical technique. There is evidence from our results that an increase in CAV-1 expression can differentiate non-metastatic cancer from normal, but this requires a more extensive study. Laser capture microdissection to isolate cancer cells would enable clarification of the expression variation in future studies.

## Conclusions

With the incidence of CRC increasing worldwide at an alarming rate, it is important to discover patient-specific panel of prognostic factors and biomarkers for early detection and treatment. Proteomics provides the opportunity to screen changes in different tissues with pathologically altered proteome compared to the normal. Non-malignant and malignant tissues were selected to determine subtle changes at molecular level bringing about major shifts in disease state.

Despite genetic variations, there are proteins which are still common among colon cancer patients. Identifying the pool of differentially expressed proteins can be of meaningful significance in understanding the disease establishment. Our preliminary study has demonstrated the ability to undertake extensive quantitative proteomics analysis of individual small colon cancer biopsies (averaging 5 mm^3^) and gain an understanding of anatomically-specific disease progression. A number of proteins were up-regulated or down-regulated through stages of the disease, were associated with well-established characteristics of cancer progression and represent targets for further investigation. An ability to validate a known target in caveolin-1, served only to confirm seemingly contradictory evidence of increase and decrease in CRC that has been reported previously. In this study, we demonstrated that levels were stage-dependent, but also varied significantly from patient-to-patient. Nevertheless, the results have provided scope to explore new solutions in population profiling, such as microarray assays that will generate diagnostics for challenging environments such as those found in developing countries.

## Supplementary information

**Additional file 1: Table S1.** Patient clinicopathological data **(a)** Patient cohort for proteomics analysis **(b)** Patient cohort for Western blot analysis.

**Additional file 2: Table S2.** Complete set of proteins identified by proteomics analysis.

**Additional file 3: Figure S1.** STRING analysis of major proteins clusters responding to colon cancer progression (i) Increased in all stages (III) (ii) Decreased in all stages (DDD) (iii) Increased only in late metastatic stage (UUI) (iv) Decreased only in late metastatic stage (UUD) (v) Increased only in benign disease (NAP) (IUU) (vi) Decreased only in benign disease (NAP) (DUU).

**Additional file 4: Figure S2.** Extended studies of CAV-1 and MMP-9 by Western blotting NC: non-cancer normal colon lining, NAP: non-adenomatous colon polyp, CC NM: colon cancer (non-metastatic), CC M: colon cancer (metastatic) E: Endoscopy patient S: Surgery patient.

**Additional file 5: Table S3.** Calculated band intensities for **(a)** CAV-1 and **(b)** β-actin after background subtraction (c) Student *t Test.*

## Data Availability

We confirm that all the associated data to this study is available and can be produced at request. Most of the data is submitted as supporting material.
