# Supplementary material for: Proteomics analysis of colon cancer progression
Source: Clin Proteomics. 2019 Dec 28;16:44. doi: 10.1186/s12014-019-9264-y (PMC6935225; doi:10.1186/s12014-019-9264-y)
Supplement: Supplementary file 3 — Additional file 3: Figure S1. STRING analysis of major proteins clusters responding to colon cancer progression (i) Increased in all stages (III) (ii) Decreased in all stages (DDD) (iii) Increased only in late metastatic stage (UUI) (iv) Decreased only in late metastatic stage (UUD) (v) Increased only in benign disease (NAP) (IUU) (vi) Decreased only in benign disease (NAP) (DUU). [file 12014_2019_9264_MOESM3_ESM.docx]

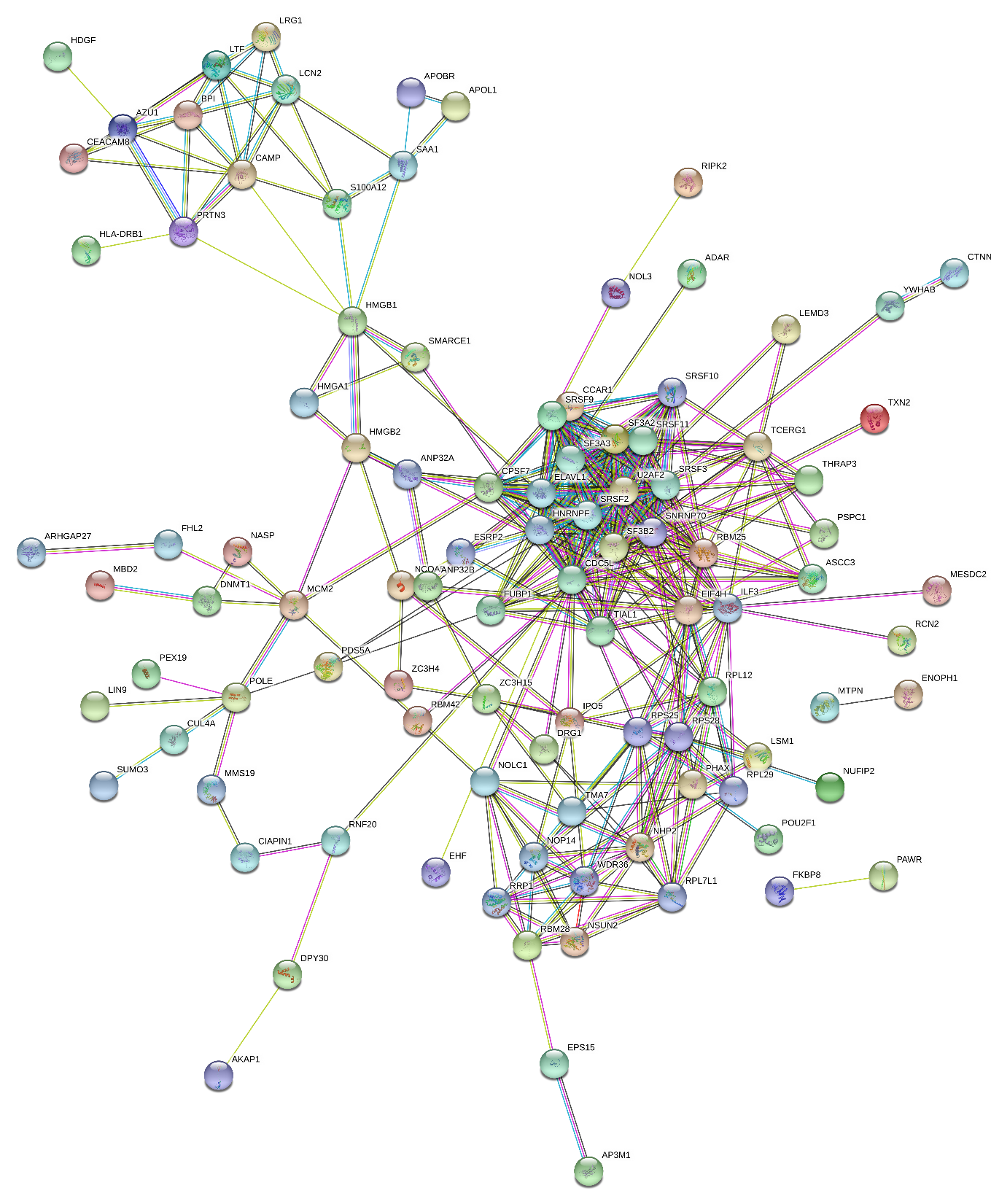


cell division cycle/

RNA processing

(i)

Increased in all (III)

Metabolism of RNA

FDR = 6.12E-15

chromatin remodelling

epigenetic modifications

Immune response


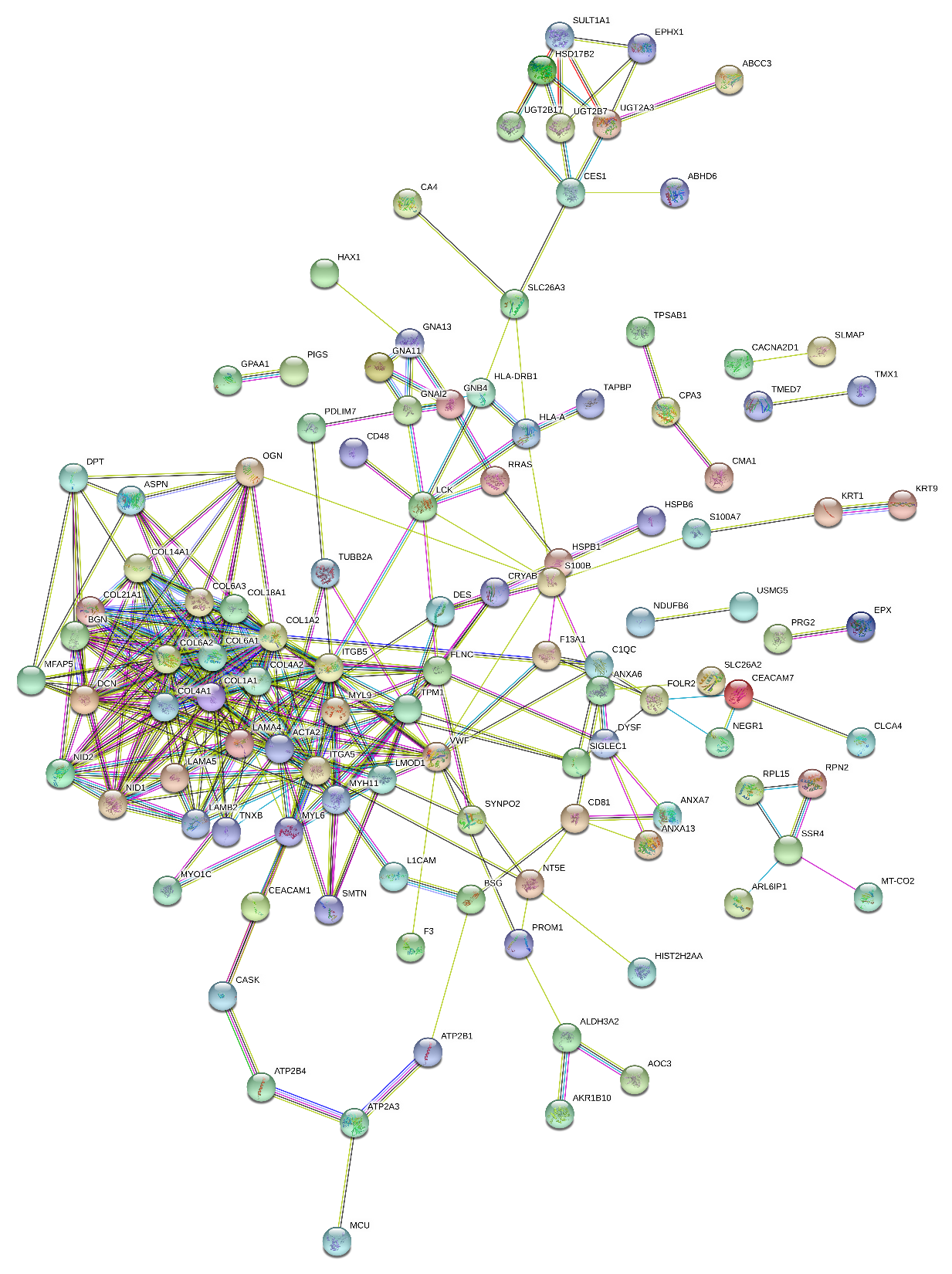


extracellular matrix

xenobiotic metabolism and transport

G-protein linked transmembrane signalling

(ii)

Decreased in all (DDD)

Extracellular matrix organisation

FDR = 8.86E-20


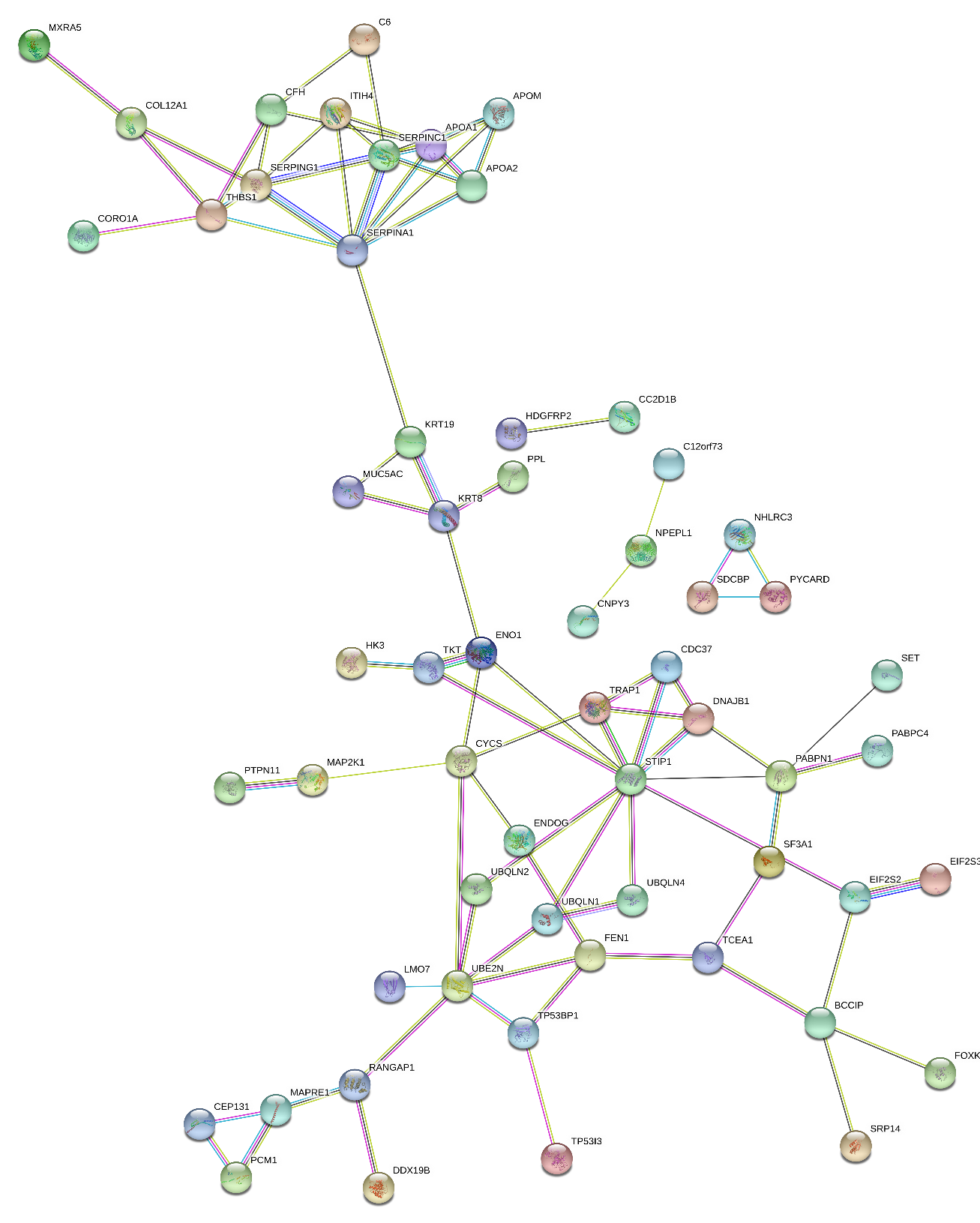


ubiquitin-associated protein turnover/ LC3-associated autophagy

immune response/ cell motility

HSP90/HSC70 associated complexes

(iii)

UUI

Innate Immune System

FDR = 3.47 E-05

(iv)

UUD

The citric acid (TCA) cycle and respiratory electron transport

FDR = 3.1 E-04


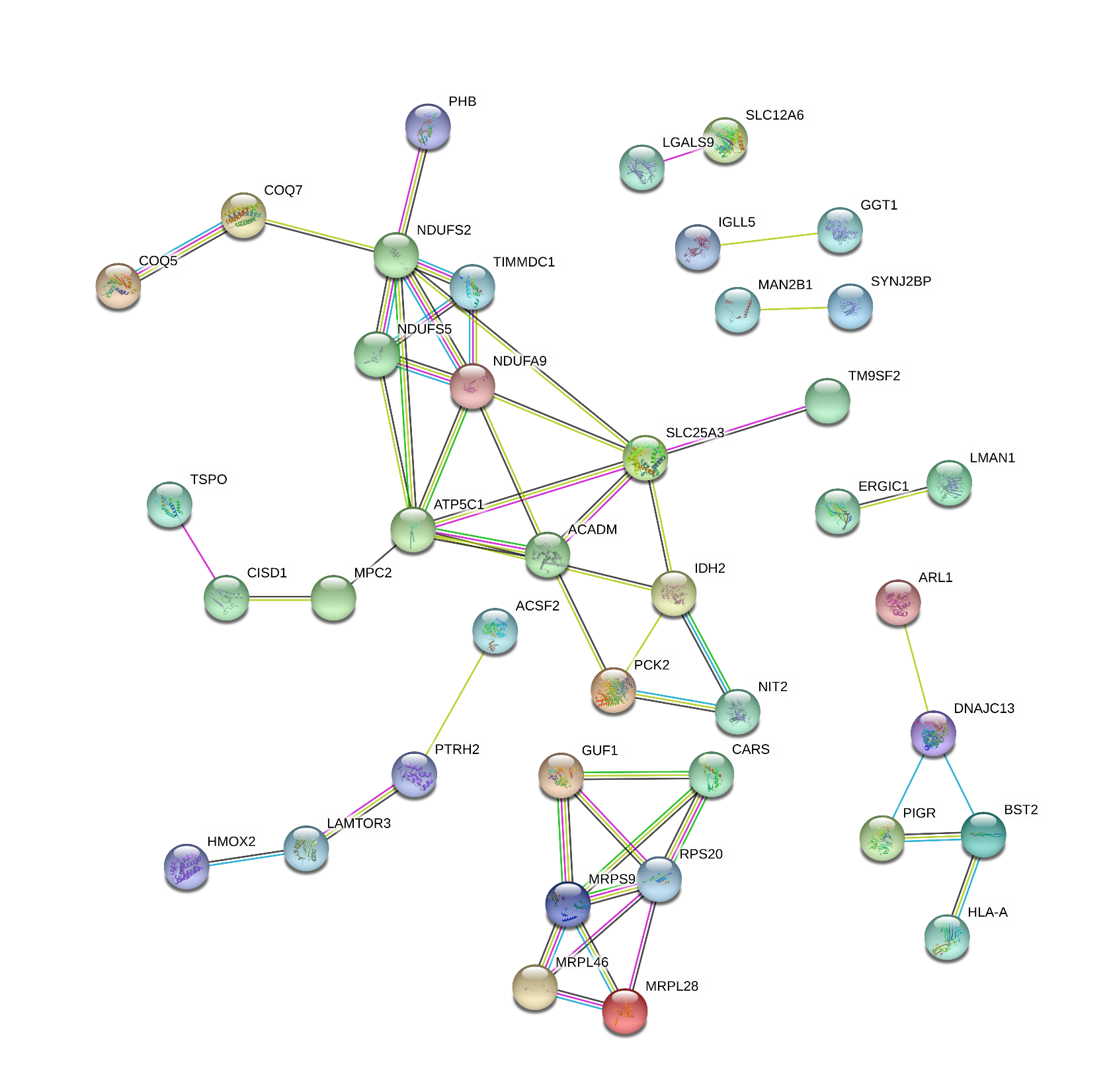


mitochondrial function

(v)

IUU

Eukaryotic Translation Elongation

FDR = 8.13E-25


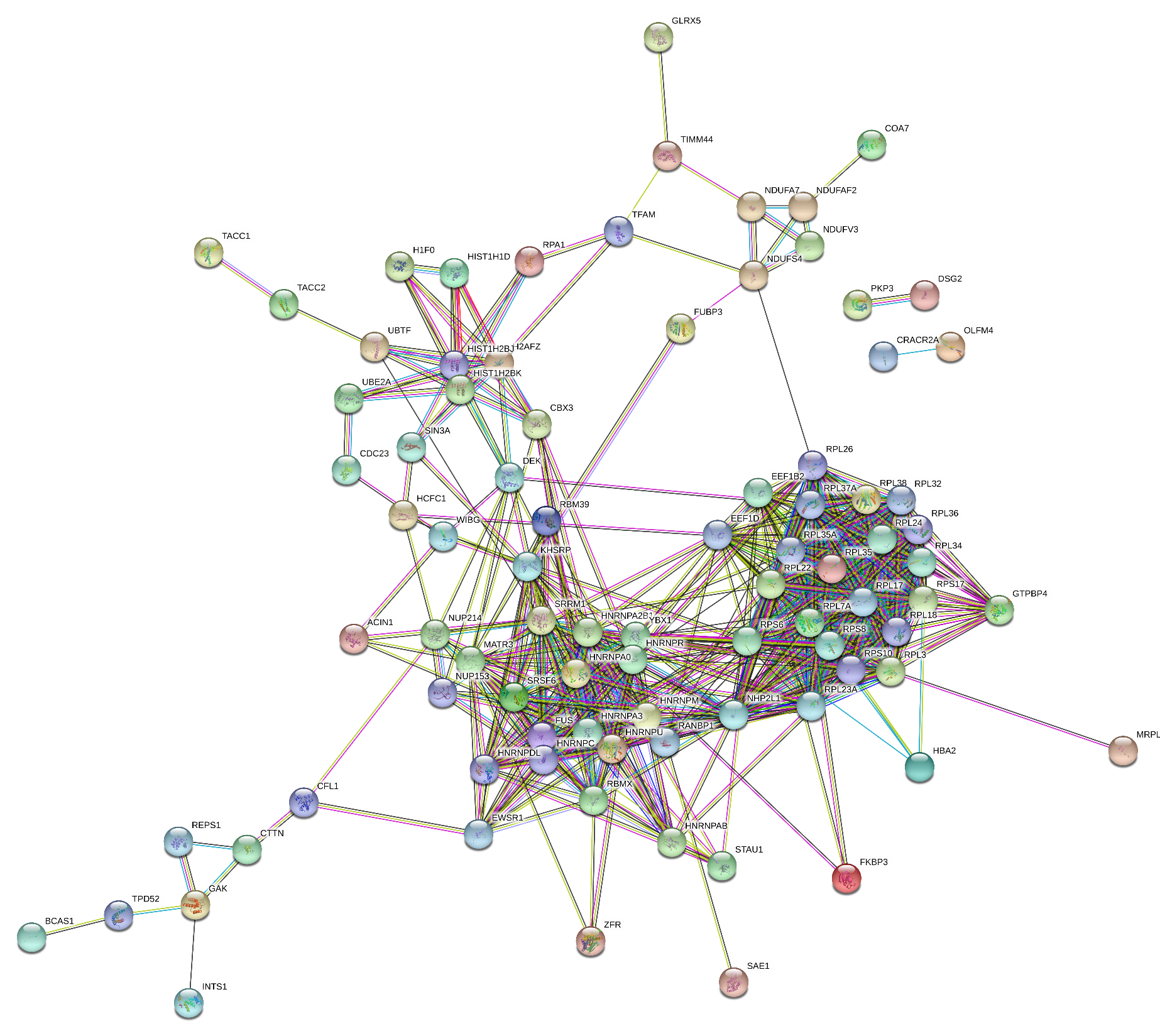


Transcriptional processing

Translational processing

(vi)

DUU

Neutrophil degranulation

FDR = 4.01E-07


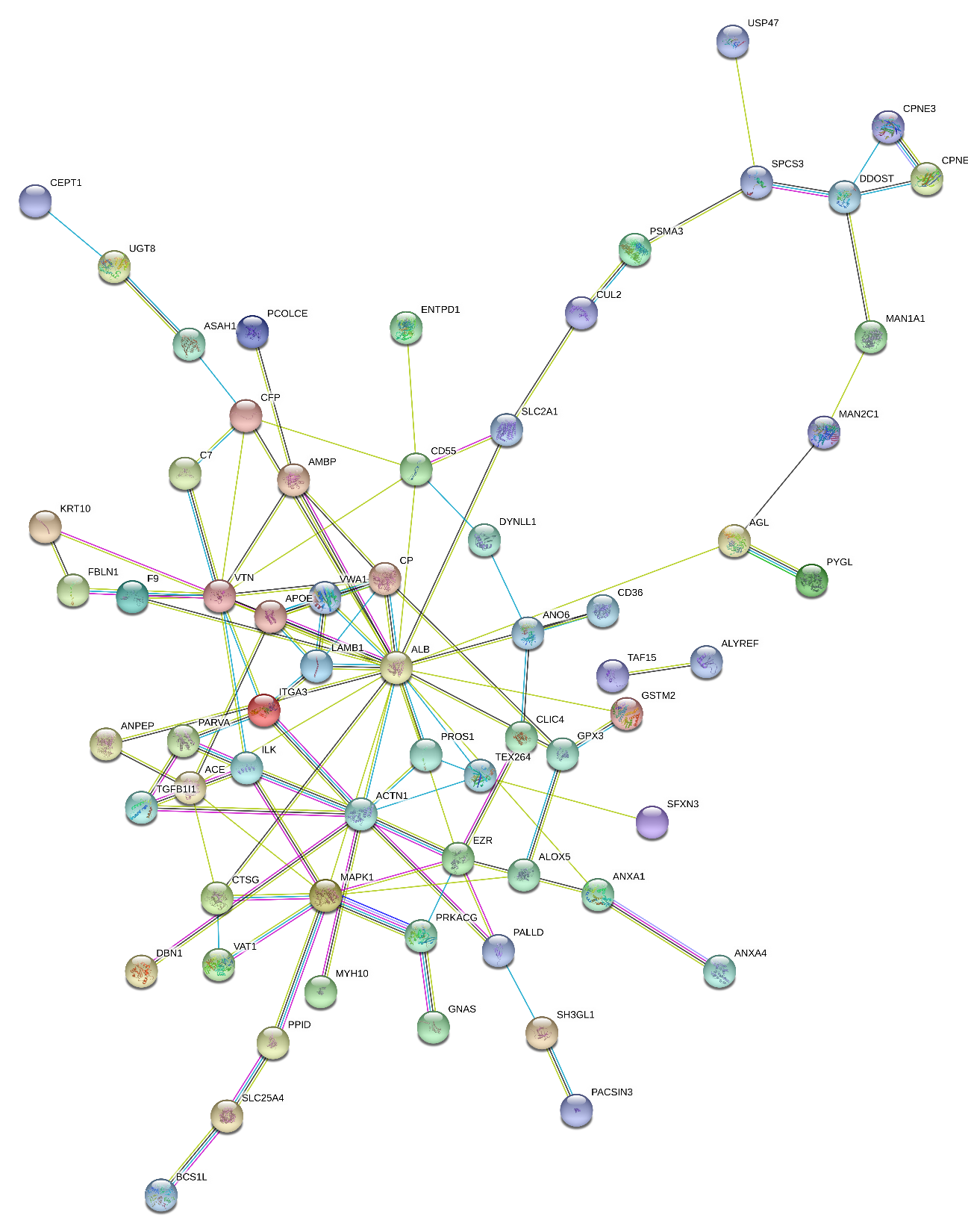


**Additional Figure S1**
