# Supplementary material for: Proteomics analysis of colon cancer progression
Source: Clin Proteomics. 2019 Dec 28;16:44. doi: 10.1186/s12014-019-9264-y (PMC6935225; doi:10.1186/s12014-019-9264-y)
Supplement: Supplementary file 4 — Additional file 4: Figure S2. Extended studies of CAV-1 and MMP-9 by Western blotting NC: non-cancer normal colon lining, NAP: non-adenomatous colon polyp, CC NM: colon cancer (non-metastatic), CC M: colon cancer (metastatic) E: Endoscopy patient S: Surgery patient. [file 12014_2019_9264_MOESM4_ESM.docx]

**Additional Figure S2**
