# Supplementary material for: Proteomics analysis of colon cancer progression
Source: Clin Proteomics. 2019 Dec 28;16:44. doi: 10.1186/s12014-019-9264-y (PMC6935225; doi:10.1186/s12014-019-9264-y)
Supplement: Supplementary file 5 — Additional file 5: Table S3. Calculated band intensities for (a) CAV-1 and (b) β-actin after background subtraction (c) Student t Test. [file 12014_2019_9264_MOESM5_ESM.docx]

**Additional Table S3**

**a** **b** **c**
